# Development of an integrated injury prevention and rehabilitation model for rugby and long-distance running: A qualitative consensus approach

**DOI:** 10.17159/2078-516X/2026/v38i1a23613

**Published:** 2026-03-15

**Authors:** S Ferreira, L Lategan, E Verhagen

**Affiliations:** 1 Department of Sport and Movement Studies, University of Johannesburg, Amsterdam UMC,Amsterdam, Netherlands; 2 Department of Public and Occupational Health, Amsterdam UMC,Amsterdam, Netherlands

**Keywords:** sports injury prevention, rehabilitation, athlete health, multidisciplinary

## Abstract

**Background:**

Sports injuries remain a significant challenge to athletes' health, performance, and long-term participation, particularly in high-contact and endurance sports. Existing prevention frameworks often lack sport-specific adaptability and integration with rehabilitation.

**Objectives:**

This study aimed to develop and refine an Integrated Injury Prevention and Rehabilitation Model (IIPRM) through a qualitative, multi-phase consensus process.

**Methods:**

A three-stage, sequential qualitative design was used. Stage 1 was a scoping review of injury risks and prevention strategies in rugby and long-distance running to identify core concepts for the model. Stage 2 used a modified e-Delphi with experts (n=22; three rounds), with consensus predefined as ≥75% agreement; panel retention was 100% across Rounds 1–3 and 18.2% attrition in Round 4. Stakeholder focus groups (n=10) were used to refine clarity, feasibility, and contextual relevance. Stage 3 combined the final fourth Delphi round and focus-group feedback to build consensus and validate the model.

**Results:**

Across Delphi rounds, 82% of items reached the ≥75% consensus threshold by Round 2, with remaining items achieving consensus in Round 3. The final IIPRM is a cyclical, athlete-centred framework integrating primary and secondary prevention through education, pre-participation screening, risk-targeted interventions, functional reintegration, and continuous monitoring. Stakeholder input prompted the inclusion of psychological readiness screening, enhanced recovery education, and seasonal alignment of interventions.

**Conclusion:**

The IIPRM provides a conceptually practical and adaptable approach to injury prevention and rehabilitation, positioned to facilitate interdisciplinary collaboration and long-term athlete health. As the model has not yet been implemented or evaluated, future research should examine its feasibility, effectiveness, and transferability across sports.

Preventing sports-related injuries is critical to athletes' health, performance, and long-term well-being. Despite advances in injury management, injury occurrence remains a persistent challenge.^[1]^ Primary prevention strategies aim to reduce injury risk before an injury occurs, while secondary prevention addresses risk factors following an injury and is crucial for recovery and minimising re-injury.^[2,3]^

A key challenge is integrating Injury Prevention Programmes (IPPs) into routine training.^[4]^ Barriers include logistical, motivational, educational, and contextual factors. Logistical challenges involve time constraints within training sessions, where technical and conditioning work are prioritised, and limited access to equipment or facilities, particularly in low-resource settings.^[4,5]^ Motivational barriers include perceptions that IPPs are monotonous or do not directly enhance performance, reducing adherence.^[6,7]^

Effective prevention requires active engagement from all stakeholders.^[8]^ However, this depends on adequate education.^[9]^ Stakeholders include athletes, coaches, medical staff, and parents. Some coaches and medical staff lack knowledge of the efficacy and implementation of IPPs, which impedes adoption ^[10]^, while lower-level coaches often report limited knowledge and confidence in delivering IPPs.^[11]^ Even experienced coaches require ongoing education and practical support to maintain IPP use.^[4]^ Athletes may lack awareness of the purpose of IPPs and of safe return-to-sport principles.^[12]^ Although medical staff value injury prevention, only 54% of European sports medicine professionals are aware of specific IPPs, due to limited expertise and staffing.^[13]^ Parents influence youth sport participation and safety, making their education about IPPs equally important.^[14]^ Organisational culture and leadership support also strongly affect implementation.^[10]^

Systematic documentation and analysis of injuries at the team or club level can enhance prevention but require sustained effort from stakeholders.^[9]^ Emerging technologies such as wearable sensors and artificial intelligence may help streamline monitoring and inform prevention strategies.^[15]^

To achieve widespread impact, injury prevention should be embedded in daily training, supported by professional medical supervision and structured IPPs.^[16,17]^ This requires a multidisciplinary approach involving athletes, coaches, medical staff, and other stakeholders.^[17]^ This paper presents a novel injury prevention and rehabilitation model that integrates primary and secondary prevention in a cyclical process, incorporating pre-participation screening (PPS), education, risk interventions, and continuous monitoring. Using individual and group interventions, the model aims to optimise physical and psychological health, support safe return to play, and promote long-term participation.^[18]^

This study aimed to develop and refine an Integrated Injury Prevention and Rehabilitation Model (IIPRM) for rugby and long-distance running, using a multi-phase qualitative design combining literature synthesis, expert consensus, and stakeholder feedback to ensure theoretical rigour, contextual relevance, and practical applicability.

## Methods

### Study design

This study followed a qualitative, multi-phase design to develop the IIPRM. The process comprised three sequential stages: (1) evidence synthesis, (2) expert and stakeholder input, and (3) consensus building and validation. Ethical clearance was granted by the University of Johannesburg Faculty of Health Sciences Research Ethics Committee (REC-01-120-2022) and Higher Degrees Committee (HDC-01-24-2021). All participants provided written informed consent.

#### Stage 1: Evidence synthesis

**Fig. 1:**
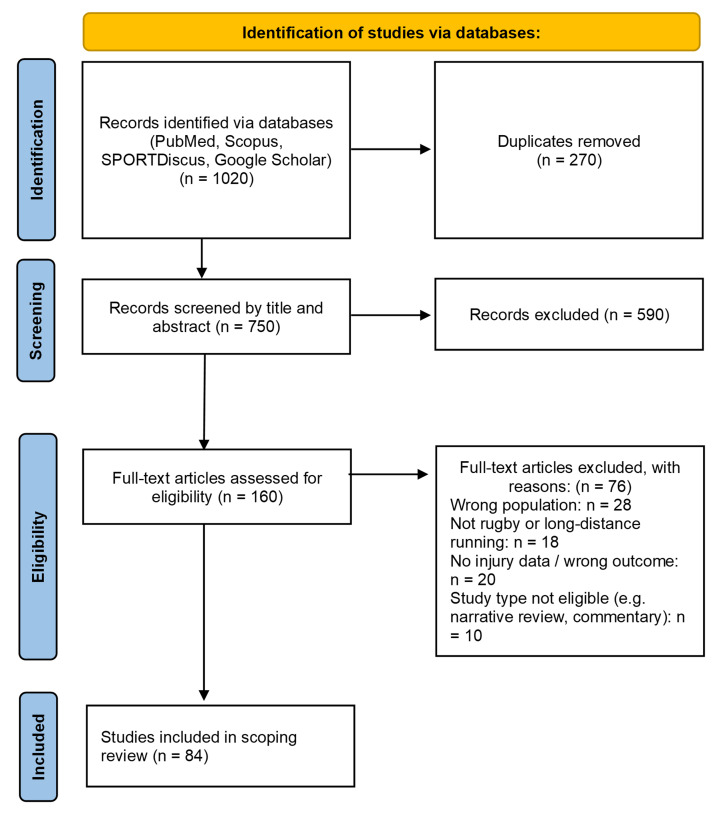
PRISMA-ScR flow diagram of study identification, screening, eligibility assessment, and inclusion for the scoping review (n = 84 included studies)

A scoping review was conducted to identify injury prevalence, risk factors, and prevention strategies in rugby and long-distance running. These sports were selected for their contrasting injury profiles, encompassing both high-contact and endurance-related risk. The review followed the Arksey and O’Malley framework ^[19]^ with enhancements from Levac et al.^[20]^ and the PRISMA-ScR guideline ^[21]^ (Figure 1). The protocol was registered with the Open Science Framework (https://osf.io/cwyt2).

Databases searched included PubMed, Scopus, SPORTDiscus and Google Scholar, with the final search conducted in December 2024. Boolean combinations included “rugby AND injury AND (risk factors OR prevention)”, “long-distance running AND injury AND (prevalence OR risk factors OR prevention strategies)”, and “endurance running AND overuse injuries AND risk factors”. Peer-reviewed English-language articles (2000–2024) reporting injury prevalence, risk factors and/or prevention strategies were included. Reviews without primary data, case reports, and grey literature were excluded.

Duplicate records were removed before screening. Title/abstract and full-text screening were conducted independently by two reviewers using predefined criteria, with disagreements resolved by discussion. Data on injury types, modifiable (e.g., poor strength, training errors) and non-modifiable (e.g., age, sex) risk factors, and prevention strategies (e.g., neuromuscular or load management programmes) were extracted and grouped by intervention type and setting. Data extraction was cross-checked by a second reviewer. A full list of included studies (n=84) is provided in the Supplementary File. Findings informed the initial IIPRM framework.

#### Stage 2: Expert and stakeholder input

##### e-Delphi process

Expert input was collected through a modified e-Delphi process. Twenty-two purposively selected experts participated in Round 1 (biokineticists n=7; sports physicians n=5; physiotherapists n=4; coaches n=6), each with >5 years of experience in sport injury prevention and rehabilitation. Round 1 comprised open-ended questions on risk factors, prevention strategies, stakeholder roles and screening/intervention practices. Responses were analysed thematically and used to construct a structured questionnaire for Round 2.

In Round 2, participants rated item relevance on a 5-point Likert scale (1 = strongly disagree; 5 = strongly agree). Items receiving <75% agreement (“agree” or “strongly agree”) were revised and re-rated in Round 3. The ≥75% threshold aligns with established Delphi standards, and stability was supported by consistent response patterns across Rounds 2–3. Descriptive statistics were used to assess consensus levels. All rounds were administered via Google Forms. All 22 experts completed Rounds 1–3 (0% attrition).

##### Focus groups

Stakeholder perspectives were obtained through two semi-structured focus groups (n=10): athletes (n=4; two rugby, two long-distance), coaches (n=3), and sports clinicians (n=3). Purposive sampling recruited participants with sport-specific experience. Discussions examined clarity, feasibility, and contextual relevance of the draft model. Both groups were facilitated by a qualitative researcher with sports medicine experience. Focus groups were held online (Microsoft Teams), audio recorded, transcribed verbatim, and analysed using thematic analysis.^[22]^

The analysis followed Braun and Clarke’s six phases: familiarisation, generating codes, developing and reviewing themes, defining and naming themes, and producing the final report. Two researchers coded independently; discrepancies were resolved through discussion. Coding was conducted manually with cross-checking by a co-coder (inter-rater reliability [IRR] = 92%). Data sufficiency was reached after the second focus group when no new themes emerged. Researchers reflected on their professional backgrounds to minimise bias. Focus group findings informed refinement of the draft IIPRM.

#### Stage 3: Consensus and model validation

Following expert and stakeholder input, the draft IIPRM was presented to the Delphi panel for a final review (Round 4). Eighteen of the 22 experts completed Round 4, providing qualitative feedback on the visual model’s logic, completeness and interdisciplinary relevance. Round 4 was distributed via Google Forms and comprised open-ended prompts on clarity, completeness and practical utility. As Round 4 served as a qualitative refinement phase, no additional quantitative consensus metrics were applied.

Feedback from Round 4 and the focus groups was synthesised to finalise the model. Specific refinements included psychological readiness screening, enhanced recovery education, and alignment of prevention strategies with training and competition phases. Findings from all stages were triangulated by comparing scoping-review themes, Delphi consensus ratings, and stakeholder narratives. Convergent findings were prioritised for inclusion; divergent items were revisited in Round 4 for clarification and contextual adjustment. The resulting IIPRM represents a dynamic, athlete-centred, interdisciplinary framework integrating prevention and rehabilitation in a continuous cycle.

## Results

### Stage 1: Evidence synthesis

**Fig. 2:**
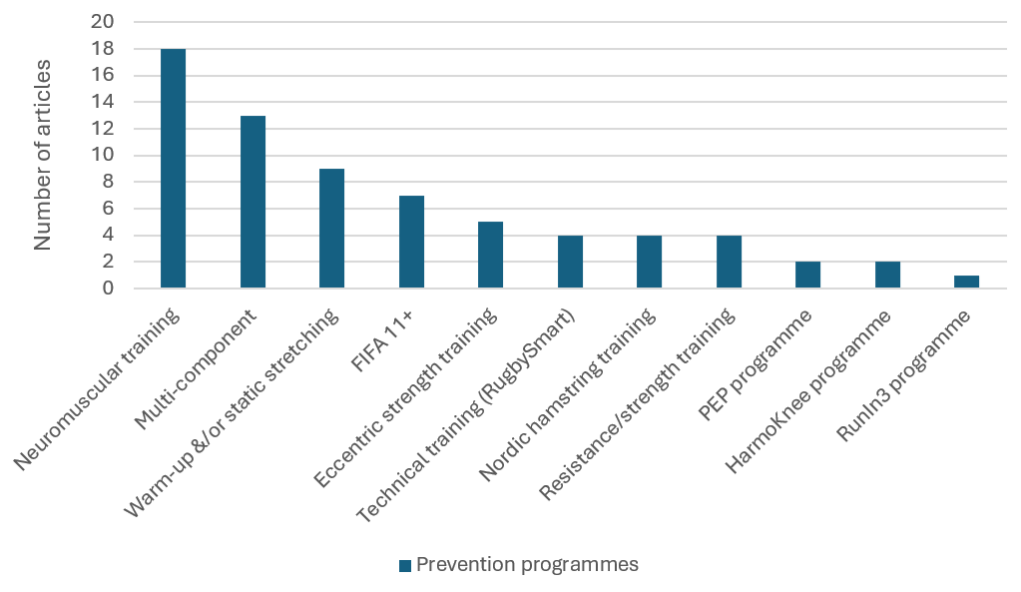
Prevention strategies (n = 11) identified in the scoping review. PEP programme, Prevent Injury and Enhance Performance programme; HarmoKnee programme, Neuromuscular warm-up programme; RunIn3 programme, Running-related injury prevention programme

The scoping review included 84 articles (rugby = 44; long-distance running = 40) and mapped evidence on injury epidemiology, risk factors and prevention strategies. Lower-limb injuries were most frequently reported, including ligament sprains, muscle strains, tendinopathies and overuse injuries. Modifiable risk factors included muscle imbalances, poor movement mechanics, training-load errors and insufficient recovery, while age, sex, anatomical variations and injury history were key non-modifiable factors. Existing prevention strategies (Figure 2) were categorised and summarised, highlighting the absence of integrated, sport-specific frameworks tailored to rugby and long-distance running. Framework papers were not excluded; rather, within the 84 included studies, no comprehensive sport-specific prevention-rehabilitation models for these sports were identified. Most publications described isolated components (e.g., warm-ups, load management, neuromuscular training) rather than integrated frameworks spanning screening, prevention, monitoring and rehabilitation. These findings informed the preliminary IIPRM structure.

### Stage 2: Expert and stakeholder input

#### e-Delphi (Rounds 1–3)

**Fig. 3:**
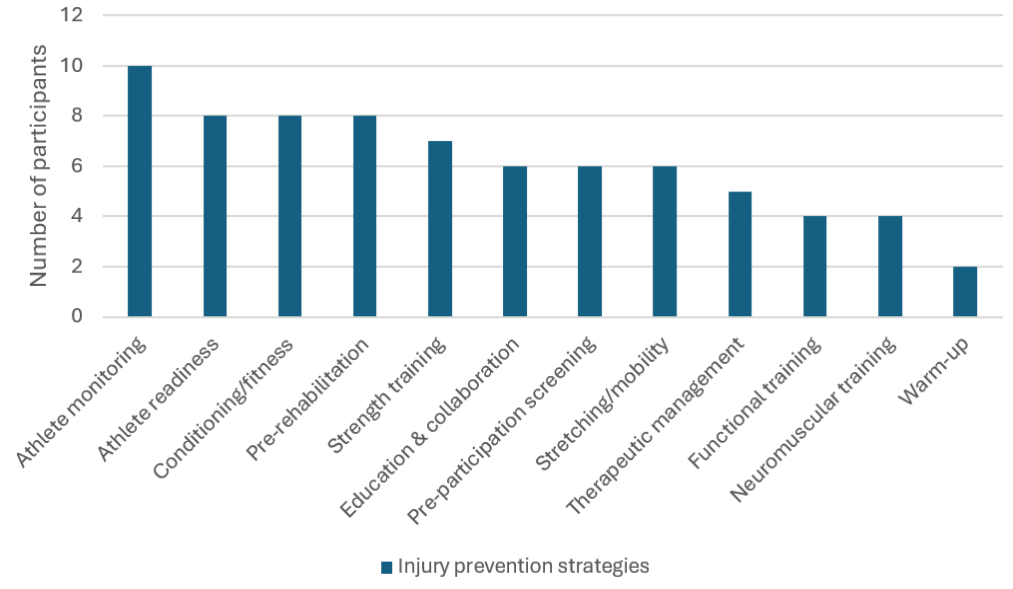
Injury prevention strategies (n = 12) identified by the experts during the Delphi process

Twenty-two experts participated in Round 1 and provided rich qualitative input on key model components. The sample was 73% male, with 59% holding a master’s or PhD qualification. The mean age was 39 years (range: 30–56), and the mean professional experience was 12.7 ± 4.5 years (Table 1). Although experts were selected across professions, some held overlapping roles. Risk factors were categorised as modifiable or non-modifiable and 12 prevention strategies were proposed (Figure 3). Experts emphasised the relevance of screening protocols, stakeholder education and targeted intervention.

In Round 2, 82% of participants agreed with the proposed elements of the model (≥75% threshold reached). Across all items, 82% reached the ≥75% consensus threshold by Round 2, with the remaining items achieving consensus after revision in Round 3. Items that did not reach consensus were revised and re-presented in Round 3, resulting in final agreement and greater clarity of key terms and concepts. All 22 experts completed Rounds 1–3 (0% attrition).

#### Stakeholder focus groups

Two focus groups (n = 10) were conducted to evaluate the clarity and contextual relevance of the draft IIPRM. Thematic analysis revealed four themes:

- *Practical applicability:*
  The cyclical and modular design was considered useful and adaptable to sport-specific contexts.
*‘I like that the model isn’t a straight line. Training and injury don’t happen in neat stages, so the cycle makes more sense.’ (Athlete 2)*
*‘The cyclical setup is really practical. We can adapt it to where the athletes are in their season, without having to redo the whole plan.’ (Coach 1)*
- *Role clarity and stakeholder engagement:*
  Clear descriptions of stakeholder roles were viewed as a strength, though uncertainty remained about the timing of involvement in settings lacking multidisciplinary structures.
*‘I appreciate how the model spells out who does what, the only thing I’m unsure about is when each person should step in.’ (Clinician 1)*
*‘It’s helpful that the model shows when each staff member should step in. The challenge is that our team does not have all those people available at all times.’ (Athlete 1)*
- *Barriers to implementation:*
  Time, resource and personnel constraints were commonly cited as challenges to implementation.
*‘Resource limitations are a big issue. We can’t always run every screening or monitoring step because the personnel just aren’t available. A potential solution would be through the education workshops, to equip current staff to do this.’ (Coach 2)*
*‘It would be great to follow the whole process, but our team doesn’t have all the staff needed, so some parts may be difficult to do.’ (Coach 1)*
- *Feedback-informed adaptations:*
  Stakeholders recommended inclusion of psychological readiness screening, enhanced recovery education and alignment of workshops with seasonal demands.
*‘I think adding something to check how mentally ready they are, would help. Sometimes the athletes are physically fine but not fully confident to return.’ (Coach 1)*
*‘Psychological readiness should definitely be part of it as we see athletes who pass strength tests, but still don’t feel prepared.’ (Clinician 1)*

### Stage 3: Expert and stakeholder input

**Fig. 4:**
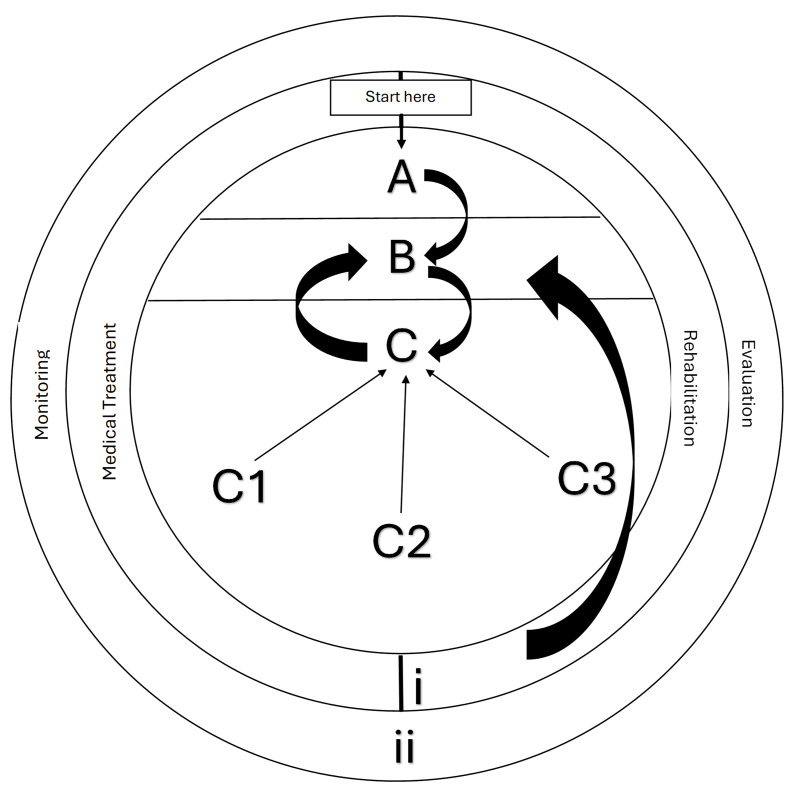
Visual representation of the Integrated Injury Prevention and Rehabilitation Model (IIPRM). Arrows illustrate the cyclical flow between education, screening, performance preparation, monitoring, early intervention, rehabilitation and return-to-play. The feedback loops indicate that athletes may re-enter any stage depending on assessment findings or rehabilitation progress.

During the final Delphi round (Round 4), 18 of the 22 participants completed the round and reviewed a visual draft of the IIPRM, confirming its suitability, clarity, and practical utility. Round 4 consisted of open-ended prompts on clarity, completeness and practical relevance. As Round 4 served as a qualitative validation step, no quantitative consensus metrics were applied in this final round, consistent with Delphi methodology for refinement phases. Minor refinements were made to terminology and visual layout. The final model (Figure 4) is a dynamic, athlete-centred framework containing two primary components: *Primary Prevention* (education, PPS and risk-targeted interventions) and *Secondary Prevention* (multi-component programmes, athlete monitoring, and sport-specific interventions). An *Injury Management and Rehabilitation* pathway surrounds the model, providing structured guidance following injury, alongside a continuous *Monitoring and Evaluation* component that tracks injury incidence, return-to-play timelines, programme adherence and athlete feedback. Stakeholder feedback confirmed that the final version is a flexible, context-sensitive system that can support both proactive and reactive approaches to athlete care.

## Discussion

This study developed a context-specific, evidence-informed IIPRM for multidisciplinary professionals working with rugby and long-distance running athletes. The model reflects the multidimensional nature of sports injury prevention, integrating primary and secondary prevention across a continuum of care. Although it builds on prior frameworks in sports medicine, it provides a structure suited to the South African healthcare context and is currently conceptually, rather than empirically, validated. Its effectiveness and real-world application require further implementation research.

The model conceptually aligns with van Mechelen et al.’s four-step framework^[23]^, which comprises injury surveillance, identification of risk factors, development of preventive interventions and evaluation. The IIPRM extends this by incorporating athlete-specific profiling and clinical reasoning early in the process, emphasising individualised assessment and practitioner-led decision-making. This is coherent with Finch’s Translating Research into Injury Prevention Practice (TRIPP) framework ^[24]^, which stresses contextual relevance and implementation.

Consistent with systems-based approaches to injury aetiology, the IIPRM includes intrinsic and extrinsic risk factors, categorised as modifiable or non-modifiable, in line with the “training–injury prevention paradox”^[25]^ Appropriate training exposure can enhance tissue capacity and reduce injury risk when managed effectively.

The dual focus on primary and secondary prevention addresses calls for a continuum-of-care approach,[26] recognising rehabilitation, reintegration and recurrence prevention as essential components of prevention strategies.^[27]^ The emphasis on education, screening and load management mirrors evidence that multi-component interventions are more effective than isolated strategies.^[28,29]^

The Delphi process strengthened clinical relevance and ensured practitioner realities were reflected. Broader frameworks such as the Haddon Matrix or socioecological models provide high-level perspectives but often lack sport-specific operational guidance.^[30]^ The IIPRM addresses this gap by offering a flexible tool for rugby and long-distance running that accommodates differing biomechanical demands within a single interdisciplinary framework, while acknowledging that transferability to other sports require further validation.

The cyclical nature of the IIPRM is central, allowing continuous movement between education, screening, preparation, monitoring, intervention and rehabilitation. Feedback loops illustrate that athletes may re-enter any stage depending on workload, symptoms, performance, or rehabilitation status. This dynamic design reflects contemporary understanding of injury risk as fluctuating across a season and requiring ongoing reassessment.

### Description of the Integrated Injury Prevention and Rehabilitation Model (IIPRM)

The IIPRM (Figure 4) consists of two core components (Primary Prevention and Secondary Prevention), encircled by an Injury Management and Rehabilitation pathway and underpinned by continuous Monitoring and Evaluation. The model is designed so that athletes can move forward or backwards between components based on changes in physical capacity, fatigue, functional demands or symptom status.

#### Primary Prevention (Sections A–C)

Primary prevention focuses on proactive measures to prevent injuries before they occur.

##### A. Education

Injury prevention begins with education delivered through sport-specific workshops for coaches, managers, strength and conditioning specialists, healthcare professionals and athletes. These sessions address injury risks, risk factors and intervention strategies tailored to club needs. They should ideally be introduced in pre- or off-season (twice per year) and revisited during the year to sustain adherence. Workshops also review injury surveillance data, update stakeholders on new evidence, and reinforce consistent implementation. Separate sessions for stakeholder groups allow targeted messaging and support. Education may also cover lifestyle factors (e.g., alcohol, tobacco, sleep, stress), goal setting and contextual challenges, encouraging engagement and early identification of potential risk contributors.

##### B. Pre-participation screening (PPS)

PPS identifies individual risk factors before athletes begin or return to sport and indicates whether medical clearance or targeted interventions are required. PPS is ideally conducted twice a year (preseason and off/post-season) by a biokineticist or sports scientist, with additional screening for injured athletes before return to play. Core components include medical and injury history, physical examination, and concussion screening, for example, using the Sport Concussion Assessment Tool (SCAT6).^[32]^

Psychological readiness is also incorporated. In the IIPRM, this refers to confidence, emotional regulation and perceived ability to perform sport-specific tasks without fear of reinjury. It can be assessed by a sports psychologist or self-report measures such as the Profile of Mood States (POMS)^[32]^ and the Psychological Readiness of Injured Athlete (PRIA) questionnaire.^[33]^ These tools, although not yet explicitly validated in South African rugby or long-distance running populations, are widely used and provide structured indicators of mental readiness. Progression decisions are informed by trends across these indicators rather than strict cut-off scores.

##### C. Risk intervention

Risk interventions can be delivered in groups (per sport) or individually (per athlete), led by biokineticists, sports scientists, strength and conditioning coaches or team coaches. Strategies are selected from components C1–C3 as a “menu” tailored to identified risks. Interventions should be implemented consistently throughout the season. Reassessment, reflected by the feedback loop from C back to B, evaluates whether risk factors have improved and whether athletes should progress, remain in intervention, or be redirected for closer monitoring.

#### Secondary Prevention (Sections C1–C3)

Sections C1–C3 are activated when emerging issues are detected through PPS or monitoring. They focus on early risk modification before or shortly after injury onset and therefore function as secondary prevention.

##### C1 – Multi-Component Programmes

Effective IPPs are multifaceted, combining preparation, strength, power, and neuromuscular control.^[28,29]^ Preparation includes warm-ups, stretching and cool-downs to prepare the musculoskeletal system and aid recovery. Strength and power work (e.g., sport-specific strength and conditioning, eccentric training, resistance training) increases tissue capacity and joint stability. Neuromuscular control components (e.g., proprioception, functional training, cross-training) target coordination, balance and movement quality while reducing overuse through movement variability. C1 is typically activated when screening or monitoring reveals deficits in strength, mobility or movement control.

##### C2 – Athlete Monitoring

Ongoing monitoring is critical in preventing injuries and supporting optimal performance. This stage encompasses:

- Training periodisation to manage load
- Ensuring adequate sleep and recovery
- Recovery techniques, such as massage or cold therapy
- Balanced nutrition and hydration
- Monitoring perceived fatigue and overall load
- Psychological well-being and mental readiness
- Assessing sport-specific readiness
- Monitoring the use of medication and supplements, the inclusion of consultation with a medical professional when needed
- Evaluating lifestyle choices that may affect health and recovery

To enhance operational clarity, practical thresholds commonly used in athlete management systems can be applied. For example, a weekly training-load increase of >10% may prompt a temporary load modification, a sustained session-rating of perceived exertion (RPE) of >7/10 may trigger an early-intervention review, and sleep <7 hours for several consecutive nights may flag insufficient recovery. These thresholds support decision-making but do not replace clinical judgement. If concerning trends emerge, athletes may be redirected to C1 for targeted interventions or to C3 when sport-specific deficits are identified.

##### C3 – Sport-Specific Interventions

C3 targets sport-specific technical and physical demands and acts as the bridge between conditioning-focused interventions and on-field performance. It includes technical skill training and return-to-play protocols customised per sport and injury type. Activation occurs when sport-specific deficits or technical risk factors are identified.

Together, C1–C3 provide a structured yet flexible framework for addressing injury risk proactively and reactively, ensuring holistic support throughout participation. Implementation in low-resource environments may require adjustments to screening frequency, staffing roles, or monitoring tools, while retaining the model's core structure.

##### Injury Management and Rehabilitation (Section i)

This outer-circle component represents tertiary management and is activated once injury has occurred. The focus shifts to structured rehabilitation, which may follow medical intervention (e.g., imaging, surgery) or conservative management. Rehabilitation should be individualised, aiming to restore function, strength, and confidence, and to address contributing risk factors.

Progression can be guided by functional benchmarks such as pain ≤2/10 during controlled activity, ≥90% limb strength symmetry, and completion of sport-specific drills without compensatory patterns. Once these criteria are met, the athlete re-enters the cycle at PPS (Stage B) to reassess residual risk and readiness. This closed-loop approach seeks to reduce reinjury risk and support long-term athlete sustainability.

##### Monitoring and Evaluation (Section ii)

Component ii monitors and evaluates the effectiveness of the IIPRM at the team or system level and supports decisions about progression, maintenance or modification of components. Evaluation should occur within phases and across the season, with key metrics summarised in Table 2 (injury incidence, severity, time loss, and return-to-play timelines).

Beyond injury data, monitoring includes programme adherence, assessed using tools such as the Sport Injury Rehabilitation Adherence Scale (SIRAS)^[34]^ or the Rehabilitation Adherence Questionnaire (RAQ).^[35]^ The choice of instrument should reflect the programme context and athlete population. Post-intervention athlete feedback, ideally anonymous, further informs which components are effective and which require adjustment, supporting continuous refinement. Overall, monitoring and evaluation function as the model’s quality-control mechanism.

### Limitations and future research

The study has several limitations. Although the Delphi and stakeholder processes supported conceptual and practical validity, this work focused on model development and did not empirically test the impact on injury outcomes. The evidence synthesis was conducted primarily within rugby and long-distance running and restricted to English-language publications, which may introduce language and publication bias. Purposive sampling for the Delphi panel may have introduced selection bias, as experts were drawn from similar sporting and professional networks. Attrition was 0% across Rounds 1–3 and 18% (4/22 experts) by Round 4, which may have influenced final qualitative refinements. The model was developed within two sports and may have limited transferability to sports with substantially different biomechanical or loading demands.

Future research should validate the IIPRM across sports and competition levels. Implementation studies are needed to evaluate feasibility, adherence and real-world effectiveness in reducing injury incidence and improving return-to-play outcomes, including in resource-constrained environments. Further refinement of the education and psychological readiness components may enhance holistic application, and future work should empirically test the cyclical structure and feedback loops.

## Conclusion

This study presents the Integrated Injury Prevention and Rehabilitation Model (IIPRM), an evidence-informed, context-specific framework designed to support multidisciplinary teams working with rugby and long-distance running athletes. By integrating injury prevention and rehabilitation within a single cyclical process, the model promotes a proactive, sustainable approach to athlete health. The IIPRM is grounded in empirical evidence, guided by expert consensus, and refined through stakeholder feedback, supporting its theoretical and conceptual applicability within the contexts studied. Future work should implement the model in real-world settings and evaluate its effectiveness in reducing injury incidence and optimising return-to-play outcomes across sports and competition levels, thereby establishing its practical utility through empirical validation.

## Supplementary Information



## Figures and Tables

**Table 1:** Socio-demographic characteristics of the Delphi expert panel

Characteristic	n (%) / mean	Range
**Sex**		
Male	16 (73%)	—
Female	6 (27%)	—
**Highest qualification**		
Master’s or PhD	13 (59%)	—
Bachelor’s degree	9 (41%)	—
**Age (years)**	Mean: 39	30–56
**Professional experience (years)**	Mean: 13	10–35
**Self-identified expertise (Round 3; n = 18)**		
Rugby expert	5 (28%)	—
Running expert	5 (28%)	—
Both rugby and running expert	4 (22%)	—
Neither running nor rugby expert	4 (22%)	—

**Table 2:** Monitoring and evaluation of injury-related metrics in athletes

Element	Monitoring tools	Evaluation method
**Number and frequency of injuries across the season**	Injury Reporting Forms Electronic Health Records (EHR) Injury Surveillance Systems	Regular data collection (weekly/monthly) Statistical analysis to track trends over the season Comparison with previous seasons
**Injury severity and type of injuries sustained**	Injury Classification Systems (e.g., Orchard Sports Injury Classification System [OSICS]) Medical Assessments	Categorization of injuries by severity (e.g., mild, moderate, severe) Analysis of injury types (e.g., sprains, fractures) Use of descriptive statistics to summarize findings
**Duration of time lost due to injury**	Injury Tracking Software Athlete Monitoring Systems	Record keeping of days lost to injury Calculation of average time lost per injury Analysis of time away from training vs. competition
**Return-to-Play rates and timelines for injured athletes**	Return-to-Play Protocols Follow-up Assessments Athlete Surveys	Tracking of return-to-play dates Comparison of expected vs. actual return timelines Surveys to assess athletes' confidence and readiness to return
