# Supplementary material for: Development of an integrated injury prevention and rehabilitation model for rugby and long-distance running: A qualitative consensus approach
Source: S Afr J Sports Med. 2026 Mar 15;38(1):v38i1a23613. doi: 10.17159/2078-516X/2026/v38i1a23613 (PMC13034982; doi:10.17159/2078-516X/2026/v38i1a23613)
Supplement: Supplementary file 1 [file 2078-516X-38-v38i1a23613-s001.pdf]

Development of an integrated injury prevention and rehabilitation model for rugby and long-distance running: A qualitative consensus approach

Supplementary Table 1. Summary of studies included in the scoping review (n = 84). *Further details of the cited articles can be requested from the corresponding author, if needed.*

Author/s	Year	Country/ countries for data collection	Sport type	Level of sport	Study design	Intervention group (n)
Hendricks, C. & Phillips, J.	2013	SA	Running	Club level	Prospective, non-experimental cohort study	50
Ellapen, T.J. et al.	2013	SA	Running	Club level	Retrospective, descriptive study	200
Badenhorst, M. et al.	2017	SA	Rugby	Provincial rugby union (clubs)	Prospective, population-based	291940
Brown, J.C. et al.	2012	SA	Rugby	Amateur & professional population	Prospective, cohort study	121663
Dunn, R.N. et al	2010	SA (Western Cape)	Rugby	Club level	Retrospective case study	27
Hillhouse, M.	2013	SA	Rugby	Varsity club level	Retrospective, descriptive study	161
Holtzhausen, L.J. et al	2006	SA	Rugby	Super 12	Prospective, cohort study	75
Millson, H.B. et al	2005	SA	Rugby	Currie Cup, club level	Retrospective, descriptive study	56
Ras, J. et al.	2014	SA	Rugby	Club level	Cross-sectional survey	77
Ras, J. et al.	2014	SA	Rugby	Club level	Cross-sectional survey	101
Schwellnuss, M.P., et al.	2014	SA	Rugby	Super Rugby (professional)	Prospective cohort study	152
Starling, L. et al	2018	SA	Rugby	Currie Cup, club level	Prospective study (injury surveillance)	132
Watson, E.D.	2014	SA	Rugby	Club level	Cross-sectional, descriptive study	100
Vosloo, S.M.	2019	SA	Rugby	Club level	Cross-sectional, descriptive study	63
Schwellnus, M.P. et al.	2012	SA	Running	Ultra-marathon runners	Prospective cohort study	68462
Borges, A.R.	2020	Brazil	Running	Recreational level	Cross-sectional observational study	95
Linton, L.	2001	USA	Running	Ultra-marathon	Prospective cohort study	184
Buist, I.	2010	Netherlands	Running	Novice runners	Prospective cohort study	532
Krabak, B.J. et al.	2011	USA	Running	General population long-distance runners	Narrative review	N/A
Noakes, T.D.	2003	SA	Running	Ultra-marathon and long-distance running	Narrative review	N/A
Taunton, J.E. et al.	2003	Canada	Running	Recreational distance runners	Retrospective cohort study	844
Taunton, J.E. et al.	2002	Canada	Running	Recreational distance runners	Retrospective cohort study	2002
van Mechelen, W.	2004	Netherlands	Running	Recreational runners	Narrative review	N/A
van Middelkoop, M. et al.	2008	Netherlands	Running	Recreational runners	Prospective cohort study	532
van Middelkoop, M. et al.	2012	Netherlands	Running	Recreational runners	Prospective cohort study	169

van Poppel, D. et al.	2020	Netherlands	Running	Recreational runners	Prospective cohort study	228
Vleck, V. et al.	2010	UK	Running	Elite triathletes	Prospective cohort study	70
Warden, S.J.	2010	USA	Running	Recreational and elite runners	Narrative review	N/A
Yamato, T.P. et al.	2015	Brazil	Running	Recreational runners	Systematic review	N/A
Yamato, T.P. et al.	2013	Brazil	Running	Recreational runners	Prospective cohort study	114
Walter, S.D. et al.	2005	Canada	Running	Urban runners	Prospective cohort study	550
Schuermans, J. et al.	2017	Belgium	Running and soccer	Elite athletes	Prospective cohort study	55
Louw, Q.A. et al.	2018	SA	Running	Marathon runners	Prospective cohort study	567
Hespanhol, L.C. Jr. et al.	2013	Netherlands	Running	Recreational novice runners	Prospective cohort study	167
Hulme, A. et al.	2017	Australia	Running	Recreational and novice runners	Prospective cohort study	784
van der Worp, M.P. et al.	2015	Netherlands	Running	Recreational runners	Systematic review	N/A
Nielsen, R.O. et al.	2013	Denmark	Running	Recreational runners	Prospective cohort study	930
Ekstrand, J. et al.	2011	Sweden & other European countries	Football, Australian football, rugby, basketball	Professional level	Prospective cohort study	N/A
King, D. et al.	2019	NZ	Rugby	Rugby union, schoolboys	Prospective cohort study	304
Archibald, J.D. & Butt, J.C.	2005	Canada	Running	Middle- and long-distance runners	Prospective cohort study	50
Boufous, S. et al.	2006	Australia	Rugby	Rugby league, community club	Retrospective cohort study	1536
Brooks, J.H.M. et al.	2005	England	Rugby	Rugby union, professional	Prospective cohort study	546
Brooks, J.H.M. & Kemp, S.P.T.	2011	England	Rugby	Rugby union, professional	Prospective cohort study	1188
Chalmers, D.J. et al.	2012	NZ	Rugby	Rugby union, community	Prospective cohort study	356
Fuller, C.W. et al.	2007	England	Rugby	Rugby union, professional	Prospective cohort study	304
Fuller, C.W. et al.	2010	England	Rugby	Rugby union, professional	Prospective cohort study	319
Gianotti, S. et al.	2009	NZ	Rugby	Rugby union, community	Prospective cohort study	304
Gabbett, T.J.	2004	Australia	Rugby	Rugby league, sub-elite	Prospective cohort study	83
Gabbett, T.J.	2012	Australia	Rugby	Rugby league, professional	Prospective cohort study	35
Haseler, C.M. et al.	2010	Australia	Rugby	Rugby union, community	Prospective cohort study	250
Holtzhausen, L. et al.	2014	SA	Rugby	Rugby union, amateur club	Prospective cohort study	264
King, D.A. et al.	2014	NZ	Rugby	Rugby union, recreational	Observational analytic prospective cohort study	N/A
McIntosh, A.S. et al.	2010	Australia	Rugby	Rugby union, junior	Prospective cohort study	98
Nicola, T.L. & Jewison, D.J.	2012	USA	Running	Distance runners	Narrative review	N/A
Opar, D. et al.	2014	Australia	Running & football codes	Elite athletes	Systematic review	N/A
Orchard, J. et al.	2013	Australia	Rugby	Rugby league & union	Prospective cohort study	N/A
Palmer, D. et al.	2019	Australia	Rugby	Rugby union, school	Prospective cohort study	362

Quarrie, K.L. et al.	2001	NZ	Rugby	Rugby union, national	Prospective cohort study	N/A
Schwellnus, M.P. et al.	2014	SA	Rugby	Super Rugby (professional)	Prospective cohort study	152
Sewry, N. et al.	2021	SA	Rugby	Rugby union, high school	Prospective cohort study	1217
Tee, J.C. et al.	2018	SA	Rugby	Rugby union, professional	Prospective cohort study	41
Tucker, R. et al.	2019	SA	Rugby	Rugby union, professional	Prospective cohort study	1272
Viljoen, W. & Patricios, J.	2012	SA	Rugby	Rugby, mixed levels	Descriptive paper (programme description)	N/A
Vosloo, S.M. & Visser, G.	2015	SA	Rugby	Varsity-level	Cross-sectional descriptive study	80
Yamato, T.P. et al.	2013	Brazil	Running	Recreational runners	Prospective cohort study	114
Meeuwisse, W.H. et al.	2007	Canada	Multiple sports including rugby	Mixed	Conceptual paper (ecological model)	N/A
Bahr, R. & Holme, I.	2003	Norway	Multiple sports including running	Mixed	Prospective cohort study	N/A
Finch, C.F.	2006	Australia	Multiple sports including rugby & running	Mixed	Conceptual framework paper	N/A
Emery, C.A.	2003	Canada	Rugby	Rugby referees	Prospective cohort study	104
Emery, C.A. et al.	2007	Canada	Rugby	Youth rugby union	Cluster randomised controlled trial	N/A
Hrysomallis, C.	2009	Australia	Multiple sports including rugby & running	Mixed	Systematic review	N/A
Myklebust, G. et al.	2003	Norway	Handball (included as IPP comparator)	Elite	Prospective cohort study	N/A
Hewett, T.E. et al.	2005	USA	Multiple sports	Female athletes	Prospective cohort study	N/A
Bahr, R.	2016	Norway	Multiple sports	Mixed	Commentary/conceptual paper	N/A
Meeuwisse, W.H. et al.	2016	Canada	Multiple sports	Mixed	Conceptual framework paper	N/A
Verhagen, E. & van Mechelen, W.	2010	Netherlands	Multiple sports including running	Mixed	Prospective controlled trial	N/A
Sugimoto, D. et al.	2012	USA	Multiple sports	Youth athletes	Meta-analysis	N/A
Thacker, S. et al.	2003	USA	Multiple sports	Mixed	Systematic review	N/A
Saleh, W. et al.	2017	UK	Multiple sports	Mixed	Systematic review & meta-analysis	N/A
Sewry, N. et al.	2017	SA	Rugby	Youth rugby union	Systematic review	N/A
Reeser, J. et al.	2006	USA	Volleyball (used for IPP context)	Elite	Systematic review	N/A
Sinibaldi, K. & Smith, D.	2007	USA	Multiple sports	Mixed	Descriptive research study	N/A
Stojanovic, M. et al.	2012	Serbia	Multiple sports	Mixed	Systematic review	N/A
Vallio, C. et al.	2021	Australia	Multiple sports	Mixed	Qualitative study	10
